# Incidence and Predictors of Mortality Among Preterm Neonates Admitted to Jimma University Medical Center, Southwest Ethiopia: a Retrospective Follow-Up Study

**DOI:** 10.3389/ijph.2024.1606897

**Published:** 2024-07-04

**Authors:** Temesgen Mohammed Toma, Hailu Merga, Lamessa Dube

**Affiliations:** ^1^ Department of Public Health Emergency Management, South Ethiopia Region Public Health Institute, Jinka, Ethiopia; ^2^ Department of Public Health, Arba Minch College of Health Sciences, Arba Minch, Ethiopia; ^3^ Department of Epidemiology, Institute of Health, Jimma University, Jimma, Ethiopia

**Keywords:** incidence, mortality, preterm, neonate, predictors

## Abstract

**Objective:**

This study aimed to assess incidence and predictors of mortality among preterm neonates in Jimma University Medical Center, Southwest Ethiopia.

**Methods:**

A retrospective follow-up study was conducted among 505 preterm neonates admitted to the Neonatal Intensive Care Unit of Jimma University Medical Center from 01 January 2017, to 30 December 2019. Data were collected from medical records using a data collection checklist. Data were entered into Epi-Data 3.1 and analyzed with STATA 15. Cox-regression analysis was fitted to identify predictors of preterm neonatal mortality. Variables with *p*-value <0.05 were declared a statistical significance.

**Result:**

The cumulative incidence of preterm neonatal death was 25.1%. The neonatal mortality rate was 28.9 deaths (95%CI: 24.33, 34.46) per 1,000 neonate-days. Obstetric complications, respiratory distress syndrome, neonatal sepsis, perinatal asphyxia, antenatal steroid exposure, gestational age at birth, and receiving kangaroo-mother care were predictors of preterm neonatal mortality.

**Conclusion:**

Preterm neonatal mortality rate was high. Hence, early detection and management of obstetric and neonatal complications, use of antenatal steroids, and kangaroo-mother care should be strengthened to increase preterm neonatal survival.

## Introduction

Preterm birth, births earlier than 37 weeks of gestational age, is a global public health priority that is linked with high neonatal morbidity and mortality, mainly in developing countries [1–4]. The preterm birth rate is increasing and great inequalities exist in a quality, access to care, and survival across countries [1]. The risk of dying is highest in the first 4 weeks of life for all babies, but preterm babies are acutely so and they need special care just to remain alive [1, 3].

Preterm birth affects nearly fifteen million people worldwide each year, with a rate of around 11% [1]. Prematurity is the leading cause of neonatal mortality and the second leading cause of death among children under the age of five worldwide. Prematurity is also the leading cause of multiple health risks in both the short and long term [1, 3]. Asia and Sub-Saharan Africa accounted for nearly 80% of all preterm births [5].

More than 35% of all neonatal mortality globally results from preventable and treatable preterm birth complications [3, 4, 6]. Nearly one million neonates die each year from preterm birth complications [7]. The survival chance of preterm neonates varies significantly based on where they were born. More than three-fourths (75%) of preterm babies could be saved with the feasible and cost-effective practice of quality care, and further reductions are possible with intensive neonatal care [1, 4].

The consequence of being born preterm extends beyond the neonatal period. They need proper care and treatment as they face greater risks of lifetime disability as well as a deprived quality of life [1, 7]. Moreover, mothers of preterm neonates experience significant psychological distress and families also endure substantial financial hardship [8, 9]. Prematurity is associated with higher healthcare costs, particularly within the first year after birth, suggesting that the implementation of appropriate programs and strategies to prevent premature delivery is beneficial from a medical as well as a healthcare expenses perspective [10].

Different findings identified mainly that mother and her neonate socio-demographic factors, maternal medical-related factors, and obstetric and gynecologic-related factors as the predictors of mortality among preterm neonates [11–18]. Ethiopia was one of the top ten countries with a high burden of preterm births globally. In Ethiopia, more than three hundred thousand neonates were born prematurely every year, and the rate of preterm birth was 12% [5, 19]. As a result, Ethiopia has adopted the new WHO recommendations for improving preterm neonatal outcomes in clinical practice [20]. Besides, the country has developed a national newborn and child survival strategy from 2015 to 2020 which aims to improve the survival of neonates, mainly preterm neonates, through the inclusion of high-impact life-saving neonatal interventions and intends to end all preventable newborn and child deaths by 2035 [21]. Despite these efforts, prematurity is the first leading cause of neonatal mortality and the fourth leading cause of mortality among children below the age of five in Ethiopia [22, 23]. Conversely, sustainable development goals (SGDs) three place a high priority on reducing newborn mortality, with a target of 12 neonatal deaths per 1,000 live births by 2030 [24]. Hence, prematurity should be addressed to curb neonatal death globally and attain SDGs [5].

There is a dearth of recent evidence on incidence and predictors of mortality among preterm neonates to inform programs and policies in Ethiopia, particularly in a study area. This significantly limits understanding of the extent and depth of the problem for evidence-based intervention. It is a dual agenda to prevent preterm delivery and address the survival gap of preterm neonates which necessitates comprehensive research to end the preventable deaths of neonates and children below 5 years. The study will help healthcare providers to identify the main predictors of preterm neonatal mortality and intervention areas, and in the timely detection of high-risk babies to give maximum efforts for their survival. Hence, this study aimed to assess the incidence and predictors of mortality among preterm neonates admitted to neonatal intensive care unit (NICU) in Jimma University Medical Center (JUMC) in Southwest Ethiopia.

## Methods

### Study Design, Period, and Setting

A retrospective follow-up study was conducted among preterm neonates admitted to NICU at JUMC between 1 January 2017, and 30 December 2019. JUMC is found in Jimma town 352 km away from Addis Ababa, the capital city of Ethiopia, in the southwestern part of the country. JUMC currently provides a range of services for approximately 15 million people. The NICU unit is one of the intensive care unit services currently in operation at the hospital which has 26 neonatal and 16 kangaroo-mother care beds. The unit also has 20 radiant warmers, four continuous positive airway pressure (CPAP), six photo-therapy machines, oxygen concentrator machines, pulse oximetry, a glucometer, and neonatal resuscitation equipment. On average, nearly 1,500 neonates are admitted annually to NICU of JUMC. The functional capability of JUMC is level three NICU [25]. The data collection period was from March 11 to 20 April 2020.

### Population

The source population for this study was all preterm neonates admitted to the NICU of JUMC from 1 January 2017, to 30 December 2019. All randomly selected preterm neonates admitted to the NICU of JUMC from 1 January 2017, to 30 December 2019, and fulfilling the eligibility criteria were the study population. All alive-born preterm neonates at admission who were registered on the neonatal registry book from 1 January 2017, to 30 December 2019, in the NICU of JUMC were included in the study. However, preterm neonates with incomplete information on medical records regarding outcome status, a time when neonates were admitted to NICU, and a time when death or censoring occurred were excluded.

### Sample Size Determination and Sampling Procedure

The sample size was determined for survival analysis by considering preterm neonates who have jaundice using STATA Version 15 statistical software based on the following assumptions: 5% level of significance (α) (two-sided), 80% power, adjusted hazard ratio of 1.62 for preterm neonates who have jaundice, overall probability of preterm neonatal death (d) of 0.288 [15], and 0.5 variabilities of covariates of interest. It was assumed that no subjects were anticipated to withdraw from the follow-up, and a 10% contingency was added for incomplete records. Hence, the total sample size for this study was 516.

The medical registration number (MRN) of preterm neonates over three 3-year period from 1 January 2017, to 30 December 2019, was taken from the NICU logbook to create a sampling frame. A total of 957 preterm neonates were admitted to NICU. A computer-generated simple random sampling technique was employed to select 516 participants for the study as follows: The sampling frame that was created using the MRN was entered into SPSS version 25 software. Then, a 516 sample was selected randomly using the SPSS select case procedure. Medical records of preterm neonates attached to selected MRNs were reviewed, and those records that met eligibility criteria were included in the analysis.

### Study Variables

Time to death of preterm neonates was the dependent variable (death was an event and coded “1,” and censored observation coded “0”). Socio-demographic related variables such as sex of neonate, neonatal age at admission, maternal age, and residence; maternal medical condition-related variables like maternal febrile illness/disease, anemia, diabetes mellitus, and human immunodeficiency virus/acquired immunodeficiency syndrome (HIV/AIDS); obstetric and gynecologic related variables like gravidity, antenatal care visit, birth type, history of bad obstetric and/or gynecologic outcome, mode of delivery, presentation, place of delivery, antenatal steroid use, and obstetric complications; and preterm neonates related variables such as gestational age at birth, birth weight, weight class for gestational age, fifth minute APGAR score, kangaroo-mother care, initiation of breastfeeding, method of feeding, congenital malformation, temperature, respiratory distress syndrome, neonatal sepsis, perinatal asphyxia, hypoglycemia, anemia, and jaundice were independent variables.

### Data Collection Instrument, Personnel, and Procedure

Data were collected from preterm neonatal medical records and registers by three trained bachelor’s degree holder midwives and supervised by one bachelor’s degree holder senior nurse. A data collection checklist adapted from the Global Neonatal Database data collection form for the Ethiopian Neonatal Network was used to collect the data [26]. Modifications were made to the checklist based on NICU registration format, and through reviewing relevant literature. The starting point for follow-up was the first NICU admission date and followed until the last neonatal period (28th days of life), which was the endpoint of the study.

### Operational Definitions

➢ **Survival status**: An outcome of the neonate during follow-up from the medical records and is considered as “death” if neonate died during follow-up, as “lost to follow-up” if the mother or caregiver was not available and unable to reach their address. It was considered as “Withdrawal” if the mother refused the follow-up due to inconvenience, as “refereed” if the neonate was referred to other institutions for better management, and “alive” if the preterm neonate survival was assured at the last follow-up period [27].
➢ **Censored**: Preterm neonates who were alive at the end of follow-up, lost to follow-up, withdrawal, or referred to other health institutions without knowing the outcome status [27].
➢ **Survival time**: Measure of the follow-up time (in days) from the date of admission in the NICU up to the date of death, censored, or the end of the study (28th day of life) [28].
➢ **Time-to-death:** Death of a preterm neonate on a specific day in the first 28 days of life [27].

### Data Quality Assurance

Data quality was assured by the careful designing of the data collection checklist, recruiting data collectors, and supervisor who have previous experience. A preliminary chart review was done on 26 randomly selected records (5% of the sample size) before the commencement of the actual study, and relevant clarifications and amendments were taken on the checklist. Training for 2 days was given on principles of research ethics, data collection checklist, and procedures for data collectors and a supervisor. Data collectors were supervised closely by the supervisor daily throughout the data collection period.

### Data Processing and Analysis

Data were cleaned, coded, and entered into Epi-Data version 3.1, and analysis was done using STATA version 15.0. Descriptive statistics such as frequencies, percentages, summary measures, and rates were computed to describe categorical and continuous variables as supposed necessary. The incidence rate of neonatal mortality was computed by dividing the number of preterm neonates who died during the follow-up period by the total neonate-days at risk of observation. The Kaplan-Meier (KM) method was used to estimate median survival time and compare survival experience between categories of variables. Log-rank test was used to compare statistically significant differences in survival experience among groups.

The Cox proportional hazard model was used to identify predictors of time to death. A bivariable cox-proportional hazard model was fitted first, and variables with a *p*-value <0.25 in this analysis entered into the multivariable cox-proportional hazard model. To identify independent predictors of time to death, a stepwise backward likelihood ratio method was used to fit a multivariable cox-proportional hazard model. An adjusted hazard ratio with a 95% confidence interval was computed to determine the strength of the association. Variables with a *p*-value <0.05 in the final model were considered significant predictors of the time to death of preterm neonates.

**FIGURE 1:**
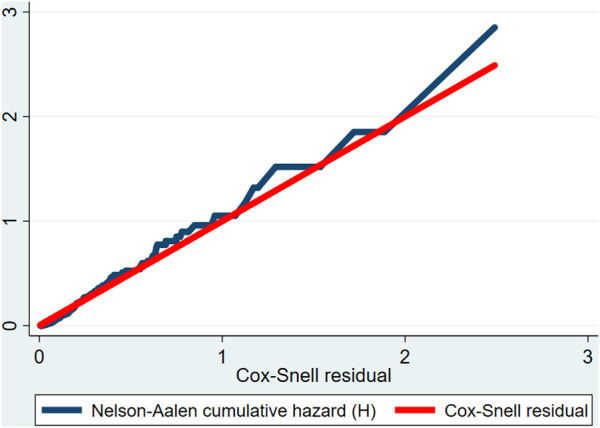
Cox-Snell residual Nelson-Aalen cumulative hazard function among preterm neonates admitted to the Neonatal Intensive Care Unit of Jimma University Medical Center, Southwest Ethiopia, 2020.

The proportional hazard assumption was checked by the Schoenfeld residual test and was satisfied (Global test X^2^ = 5.13, *p*-value = 0.92). Multicollinearity was checked by looking at the variance inflation factor (VIF) and the highest observed VIF value was 2.06, indicating that there was no multicollinearity threat. The goodness of model fitness was evaluated by using the Cox-Snell residual test. In this study, the Nelson-Aalen cumulative hazard function follows the 45° diagonal line very closely, indicating that it almost has an exponential distribution with a hazard rate of one. Hence, for the residual test, it was possible to conclude that the final model fits the data very well (Figure 1).

## Results

A total of 516 preterm neonate medical records were reviewed, and 505 records that met eligibility criteria were included in the analysis with a response rate of 97.8%. Eleven medical records which did not fulfil the eligibility criteria were excluded. From excluded records, 6 records had incomplete data on outcome status, 3 records had an unknown date when the outcome of interest happened, and 2 records had an unknown date of admission to NICU (Supplementary Material S1).

### Socio-Demographic Characteristics

Near to nine-tenth of neonates, 433 (85.7%), had less than 24 h of age at admission and more than half, 279 (55.2%), of them were males. The median age of the mother was 27 years (an interquartile range (IQR) of 8 years). Most mothers of the neonates, 398 (78.8%), were between the ages of 20 and 34. Nearly two-thirds, 339 (67.1%), of preterm neonates were rural residents (Table 1).

**TABLE 1:** Socio-demographic characteristics of preterm neonates and their mothers at Neonatal Intensive Care Unit of Jimma University Medical Center, Jimma, Southwest Ethiopia, 2020 (N = 505).

Variable	Categories	Survival status		Total (%)
		Death N (%)	Censored N (%)	
Neonatal age at admission	<1 day 1–6 days ≥7 days	109 (25.2) 17 (26.6) 1 (12.5)	324 (74.8) 47 (73.4) 7 (87.5)	433 (85.7) 64 (12.7) 8 (1.6)
Sex of neonate	Male Female	74 (26.5) 53 (23.5)	205 (73.5) 173 (76.5)	279 (55.2) 226 (44.8)
Age of the mother (in years)	<20 20–34 ≥35	3 (30.0) 93 (23.4) 31 (32.0)	7 (70.0) 305 (76.6) 66 (68.0)	10 (2.0) 398 (78.8) 97 (19.2)
Residence	Rural Urban	87 (25.7) 40 (24.1)	252 (74.3) 126 (75.9)	339 (67.1) 166 (32.9)

### Maternal Medical, Obstetric, and Gynecologic Characteristics

Of the participants, 66 (13.1%) mothers had known or been diagnosed with a medical disease, and more than nine-tenths of the mothers, 467 (92.5%), had antenatal care visits during the current pregnancy. Almost a quarter, 124 (24.6%), of the mothers had used antenatal steroids. Nearly three-fourths, 372 (73.7%), of the mothers had spontaneous onset of labor, and the majority of the delivery, 462 (91.5%), had a cephalic presentation. Almost half, 250 (49.5%), of the mothers had an obstetric complication (Table 2).

**TABLE 2:** Maternal medical, obstetric, and/or gynecologic characteristics of a study participant in the Neonatal Intensive Care Unit of Jimma University Medical Center, Jimma, Southwest Ethiopia, 2020 (N = 505).

Variable	Categories	Survival status		Total (%)
		Death N (%)	Censored N (%)	
Known or diagnosed medical diseases	Yes No	20 (30.3) 107 (24.4)	46 (69.7) 332 (75.6)	66 (13.1) 439 (86.9)
Febrile illness or disease	Yes No	11 (33.3) 116 (24.6)	22 (66.7) 356 (75.4)	33 (6.5) 472 (93.5)
Anemia	Yes No	7 (33.3) 120 (24.8)	14 (66.7) 364 (75.2)	21 (4.2) 484 (95.8)
Others^a^	Yes No	3 (15.8) 124 (25.5)	16 (84.2) 362 (74.5)	19 (3.8) 486 (96.2)
Gravidity	I II-IV ≥V	38 (22.4) 64 (25.4) 25 (30.1)	132 (77.6) 188 (74.6) 58 (69.9)	170 (33.7) 252 (49.9) 83 (16.4)
Antenatal care visit	Yes No	108 (23.1) 19 (50.0)	359 (76.9) 19 (50.0)	467 (925) 38 (7.5)
Birth type	Single Twin	91 (25.3) 36 (24.8)	269 (74.7) 109 (75.2)	360 (71.3) 145 (28.7)
Bad obstetric and/or gynecologic history^b^	Yes No	38 (32.2) 89 (23.0)	80 (67.8) 298 (77.0)	118 (23.4) 387 (76.6)
Antenatal steroid use	Yes No	22 (17.7) 105 (27.6)	102 (82.3) 276 (72.4)	124 (24.6) 381 (75.4)
Mode of delivery	Spontaneous vaginal delivery Assisted vaginal delivery Cesarean section (C/S)	79 (23.5) 3 (14.3) 45 (30.4)	257 (76.5) 18 (85.7) 103 (69.6)	336 (66.5) 21 (4.2) 148 (29.3)
Cause of onset of labor	Spontaneous Induced C/S	89 (23.9) 12 (29.3) 26 (28.3)	283 (76.1) 29 (70.7) 66 (71.7)	372 (73.7) 41 (8.1) 92 (18.2)
Presentation	Cephalic Non-cephalic	116 (25.1) 11 (25.6)	346 (74.9) 32 (74.4)	462 (91.5) 43 (8.5)
Place of delivery	Hospital Health center Home	97 (24.9) 20 (23.3) 9 (34.6)	292 (75.1) 69 (76.7) 17 (65.4)	389 (77.0) 90 (17.8) 26 (5.2)
Obstetric complications	Yes No	75 (30.0) 52 (20.4)	175 (70.0) 203 (79.6)	250 (49.5) 255 (50.5)
Preeclampsia	Yes No	23 (28.8) 104 (24.5)	57 (71.2) 321 (75.5)	80 (15.8) 425 (84.2)
Eclampsia	Yes No	8 (32.0) 119 (24.8)	17 (68.0) 361 (75.2)	25 (5.0) 480 (95.0)
Fetal distress	Yes No	26 (32.1) 101 (23.8)	55 (67.9) 323 (76.2)	81 (16.0) 424 (84.0)
Preterm premature rupture of membrane	Yes No	20 (32.3) 107 (24.2)	42 (67.7) 336 (75.8)	62 (12.3) 443 (87.7)
Abruption placenta	Yes No	6 (18.7) 121 (25.6)	26 (81.3) 352 (74.4)	32 (6.3) 473 (93.7)
Placenta Previa	Yes No	8 (34.8) 119 (24.7)	15 (65.2) 363 (75.3)	23 (4.6) 482 (95.4)
Chorioamnionitis	Yes No	9 (32.1) 118 (24.7)	19 (67.9) 359 (75.3)	28 (5.5) 477 (94.5)
Others^c^	Yes No	10 (41.7) 117 (24.3)	14 (58.3) 364 (75.7)	24 (4.8) 481 (95.2)

^a^ Diabetes mellitus, HIV/AIDS, cardiac disease, renal disease, and STIs.

^b^ Neonatal death, stillbirth, abortion, and intrauterine fetal death.

^c^ Cord prolapse, oligohydramnios, polyhydramnios, and prolonged labor.

### Preterm Neonate-Related Characteristics

Almost four-fifths of the neonates, 399 (79%), were moderate preterm, and more than two-thirds of neonates, 349 (69%), had low birth weight. Out of the cohort, 454 (89.9%) had not initiated breastfeeding within 1 hour of birth. More than three-fourths of neonates, 397 (78.6%), were diagnosed with hypothermia followed by respiratory distress syndrome 295 (58.4%), and hypoglycemia 167 (33.1%). Kangaroo-mother care (KMC) was provided to nearly one in every ten neonates (30.7%). More than three-fifths of neonates, 59.6%, received nasal CPAP (Table 3).

**TABLE 3:** Neonatal-related characteristics of preterm neonate admitted to Neonatal Intensive Care Unit of Jimma University Medical Center, Jimma, Southwest Ethiopia, 2020 (N = 505).

Variable	Categories	Survival status		Total (%)
		Death N (%)	Censored N (%)	
Gestational age at birth (in weeks)	<28 28- <32 32- <37	14 (70.0) 34 (39.5) 79 (19.8)	6 (30.0) 52 (60.5) 320 (80.2)	20 (4.0) 86 (17.0) 399 (79.0)
Birth weight (in grams)	<1,000 1,000–1,499 1,500–2,499 ≥2,500	15 (62.5) 40 (36.7) 69 (19.8) 3 (13.0)	9 (37.5) 69 (63.3) 280 (80.2) 20 (87.0)	24 (4.8) 109 (21.6) 349 (69.0) 23 (4.6)
Weight class for gestational age	AGA SGA LGA	122 (25.3) 5 (29.4) 0 (0.0)	360 (74.7) 12 (70.6) 6 (100.0)	482 (95.4) 17 (3.4) 6 (1.2)
Fifth-minute APGAR score	<7 ≥7	41 (38.7) 86 (21.6)	65 (61.3) 313 (78.4)	106 (21.0) 399 (79.0)
Initiation of breastfeeding within 1 hour of birth	Yes No	5 (9.8) 122 (26.9)	46 (90.2) 332 (73.1)	51 (10.1) 454 (89.9)
Method of feeding	Breast sucking NGT Cup feeding	26 (18.6) 99 (28.2) 2 (14.3)	114 (81.4) 252 (71.8) 12 (85.7)	140 (27.7) 351 (69.5) 14 (2.8)
Hypothermia	Yes No	102 (25.7) 25 (23.1)	295 (74.3) 83 (76.9)	397 (78.6) 108 (21.4)
Respiratory distress syndrome	Yes No	93 (31.5) 34 (16.2)	202 (68.5) 176 (83.8)	295 (58.4) 210 (41.6)
Neonatal sepsis	Yes No	51 (33.8) 76 (21.5)	100 (66.2) 278 (78.5)	151 (29.9) 354 (70.1)
Perinatal asphyxia	Yes No	17 (42.5) 110 (23.7)	23 (57.5) 355 (76.3)	40 (7.9) 465 (92.1)
Hypoglycemia	Yes No	47 (28.1) 80 (23.7)	120 (71.9) 258 (76.3)	167 (33.1) 338 (66.9)
Anemia	Yes No	10 (29.4) 117 (24.8)	24 (70.6) 354 (75.2)	34 (6.7) 471 (93.2)
Jaundice	Yes No	45 (36.9) 82 (21.4)	77 (63.1) 301 (78.6)	122 (24.2) 383 (75.8)
Congenital malformation	Yes No	8 (36.4) 119 (24.6)	14 (63.6) 364 (75.4)	22 (4.4) 483 (95.6)
Apnea of prematurity	Yes No	8 (50.0) 119 (24.3)	8 (50.0) 370 (75.7)	16 (3.2) 489 (96.8)
Others*	Yes No	3 (10.3) 124 (26.1)	26 (89.7) 352 (73.9)	29 (5.7) 471 (94.3)
Received kangaroo mother care	Yes No	22 (14.2) 105 (30.0)	133 (85.8) 245 (70.0)	155 (30.7) 350 (69.3)
Received nasal CPAP	Yes No	85 (28.2) 42 (20.6)	216 (71.8) 162 (79.4)	301 (59.6) 204 (40.4)
Resuscitated with a bag and mask	Yes No	43 (27.0) 84 (24.3)	116 (73.0) 262 (75.7)	159 (31.5) 346 (68.5)
Received phototherapy	Yes No	36 (31.9) 91 (23.2)	77 (68.1) 301 (76.8)	113 (22.4) 392 (77.6)
Heated with radiant warmer	Yes No	98 (25.6) 29 (23.8)	285 (74.4) 93 (76.2)	383 (75.8) 122 (24.2)

^a^ Meningitis, ophthalmic neonatorum, necrotizing enterocolitis, pulmonary hypertension, HIV, exposed, meconium aspiration syndrome, hospital-acquired infection, and birth trauma.

### Incidence of Mortality Among Preterm Neonate

During the follow-up, the cumulative incidence of preterm neonatal death was 127 (25.1%). Of all deaths, 15.7% died in the first 24 h of life, and 81.1% of deaths occurred within 7 days of life. Out of the cohort, 352 (69.7%) improved and were discharged to home, 15 (3.0%) lost to follow-up, 6 (1.2%) were referred to other hospitals, and the remaining 5 (1.0%) were withdrawn from the follow-up.

A cohort contributed a total of 4,386 neonate days at risk of observation. The overall neonatal mortality rate (incidence density) was 28.9 deaths per 1,000 neonate-days (95% CI: 24.33, 34.46). The neonatal mortality rate (NMR) was 67.3 deaths per 1,000 neonate-days in the first 24 h of life (95% CI: 48.11, 94.23). Early NMR (death within 7 days of life) was 40 deaths per 1,000 neonate-days (95% CI: 33.08, 48.33); however, the late NMR was 11.7 deaths per 1,000 neonate-days (95% CI: 7.55, 18.13).

### Overall Survival Function

Preterm neonates were followed for different periods: a minimum of 1 day and a maximum of 28 days. The overall median length of follow-up was 7 (IQR = 8) days. The cumulative survival probability at the end of the follow-up was 54.94% (95% CI: 41.83, 66.27). The cumulative probability of survival at the end of the first, seventh, 14th, and 21st days was 93.27% (95% CI: 90.71, 95.14), 76.89% (95% CI: 72.73, 80.51), 71.8% (95% CI: 66.79, 76.19), and 66.96% (60.54, 72.58), respectively. The overall mean survival time was 20.42 neonate days (95% CI: 19.27, 21.56).

### Survival Function and Comparison of Survival Experience

**FIGURE 2:**
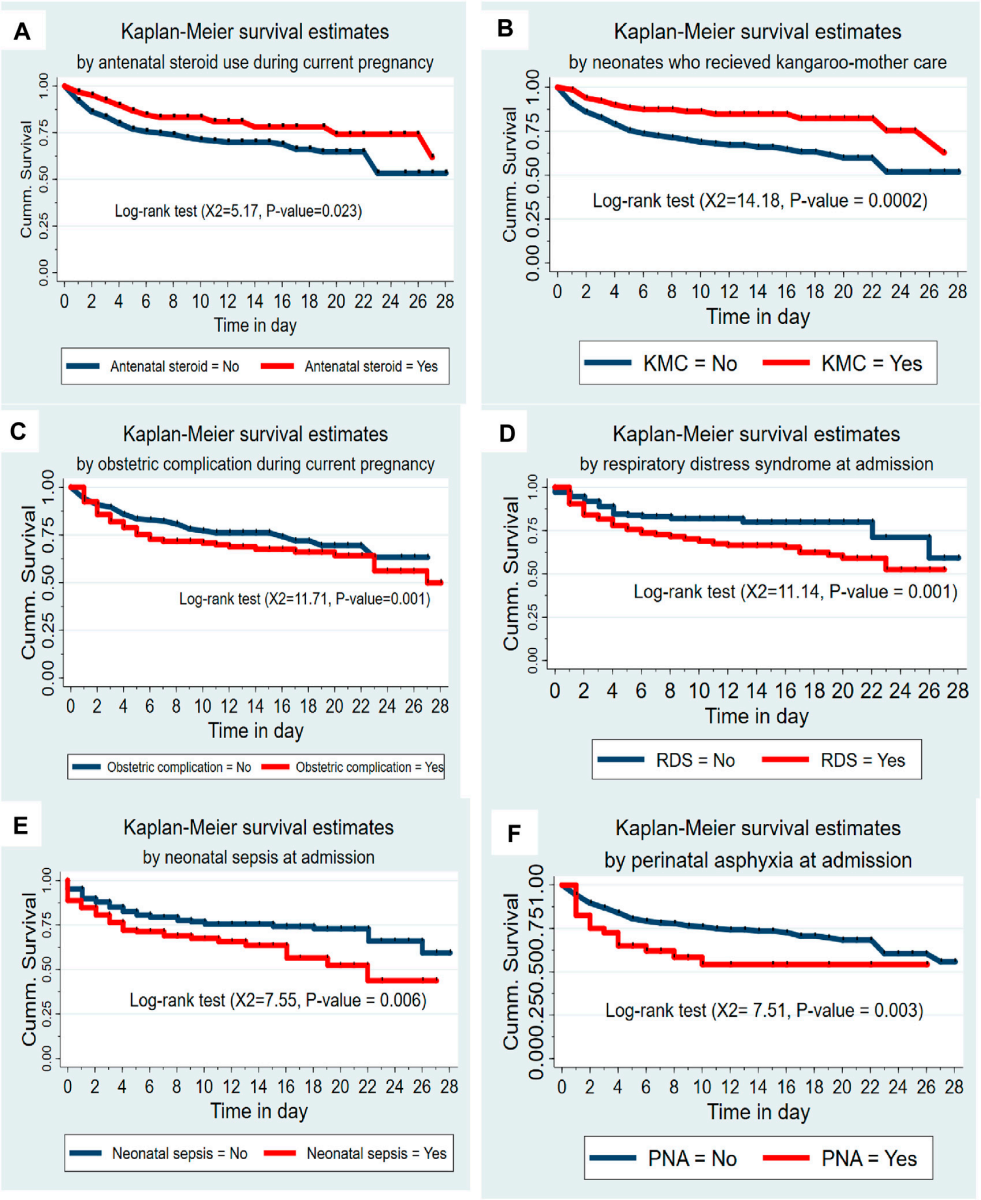
**(A)** The Kaplan-Meier survival curves by antenatal steroid use among preterm neonates admitted to the Neonatal Intensive Care Unit at Jimma University Medical Center, Southwest Ethiopia, 2020 (*n* = 505). **(B)** The Kaplan-Meier survival curves by KMC service among preterm neonates admitted to the Neonatal Intensive Care Unit at Jimma University Medical Center, Southwest Ethiopia, 2020 (*n* = 505). **(C)** The Kaplan-Meier survival curves by obstetric complication among preterm neonates admitted to the Neonatal Intensive Care Unit at Jimma University Medical Center, Southwest Ethiopia, 2020 (*n* = 505). **(D)** The Kaplan-Meier survival curves by respiratory distress syndrome among preterm neonates admitted to the Neonatal Intensive Care Unit at Jimma University Medical Center, Southwest Ethiopia, 2020 (*n* = 505). **(E)** The Kaplan-Meier survival curves by neonatal sepsis among preterm neonates admitted to the Neonatal Intensive Care Unit at Jimma University Medical Center, Southwest Ethiopia, 2020 (*n* = 505). **(F)** The Kaplan-Meier survival curves by perinatal asphyxia among preterm neonates admitted to the Neonatal Intensive Care Unit at Jimma University Medical Center, Southwest Ethiopia, 2020 (*n* = 505).

Preterm neonates born from mothers who used antenatal steroids had higher survival experiences compared to their counterparts (chi-square = 5.17, *p*-value = 0.023). Likewise, preterm neonates who received KMC had a higher survival experience than neonates who didn’t receive KMC (chi-square = 14.18, *p*-value = 0.0002). However, preterm neonates born from mothers with obstetric complications had lower survival experience than their counterparts (chi-square = 11.71, *p*-value = 0.001). Neonates having respiratory distress syndrome (RDS) had lower survival experiences than neonates without RDS (chi-square = 11.14, *p*-value = 0.001). Preterm neonates with neonatal sepsis had lower survival experiences than their complements (chi-square = 7.55, *p*-value = 0.006). Neonates with perinatal asphyxia (PNA) had lower survival experiences than their counterparts (chi-square = 7.51, *p*-value = 0.003) (Figure 2).

### Predictors of Preterm Neonatal Mortality

In the multivariable cox-regression model; antenatal steroid use, obstetric complication during the current pregnancy, an increment in gestational age at birth, receiving KMC, having RDS, neonatal sepsis, and PNA were found to be predictors for time to death of preterm neonates at *p*-value <0.05.

Preterm neonates born from mothers who used antenatal steroids during current pregnancy had 45% fewer hazard of death compared to neonates born from mothers who didn’t use antenatal steroids (AHR = 0.55; 95% CI:0.34, 0.90). Preterm neonates born from mothers with an obstetric complication had a 1.84 times higher hazard of death compared to those who were born from mothers without obstetric complication (AHR = 1.84; 95% CI: 1.20, 2.82).

As the gestational age of preterm neonates at birth increases by 1 week, the hazard of death decreases by 19% (AHR = 0.81; 95% CI: 0.75, 0.87). Preterm neonates who had RDS had 1.52 times more hazard of death than those without RDS (AHR = 1.52; 95% CI:1.01, 2.29). Preterm neonates who had neonatal sepsis had about 1.71 times greater hazard of death than neonates without neonatal sepsis (AHR = 1.71; 95% CI: 1.18, 2.49). Preterm neonates who had PNA had 2.44 times more hazard of death compared to those neonates without PNA (AHR = 2.44; 95% CI: 1.33, 4.49). Preterm neonates who received KMC had 52% lesser hazard of death as compared to their counterparts (AHR = 0.48; 95% CI: 0.30, 0.77) (Table 4).

**TABLE 4:** Bivariable and multivariable cox-regression model for predictors of preterm neonatal mortality in Neonatal Intensive Care Unit of Jimma University Medical Center, Jimma, Southwest Ethiopia, 2020 (N = 505).

Variables	Survival status		CHR [95% CI]	*p*-value	AHR [95% CI]	*p*-value
	Death n (%)	Censored n (%)				
Age of the mother						
<20 years 20–34 years ≥35 years	3 (30.0) 93 (23.4) 31 (32.0)	7 (70.0) 305 (76.6) 66 (68.0)	1.50 [0.47–4.73] 1 1.43 [0.95–2.15]	0.49 0.087	3.04 [0.88–10.54] 1 1.11 [0.73–1.69]	0.079 0.62
Antenatal care visit during the current pregnancy						
Yes No	108 (23.1) 19 (50.0)	359 (76.9) 19 (50.0)	1 2.25 [1.38–3.67]	0.001	1 1.29 [0.75–2.23]	0.35
History of bad obstetric and/or gynecologic outcomes						
Yes No	38 (32.2) 89 (23.0)	80 (67.8) 298 (77.0)	1.33 [0.91–1.95] 1	0.14	0.91 [0.60–1.34] 1	0.64
Antenatal steroid use during the current pregnancy						
Yes No	22 (17.7) 105 (27.6)	102 (82.3) 276 (72.4)	0.59 [0.38–0.94] 1	0.028	0.55 [0.34–0.90] 1	0.018
Place of delivery						
Hospital Health center Home	97 (24.9) 20 (29.0) 9 (34.6)	292 (75.1) 69 (71.0) 17 (65.4)	1 1.04 [0.65–1.67] 1.68 [0.84–3.33]	0.87 0.14	1 1.64 [0.95–2.82] 2.07 [0.99–4.32]	0.074 0.051

Obstetric complications during the current pregnancy						
Yes No	75 (30.0) 52 (20.4)	175 (70.0) 203 (79.6)	1.44 [1.01–2.05] 1	0.045	1.84 [1.20–2.82] 1	0.005
Gestational age at birth (in a week)			0.83 [0.77–0.89]	<0.001	0.81 [0.75–0.87]	<0.001
Birth weight (in grams)						
<1,000 1,000- <1,500 1,500- <2,500 ≥2,500	15 (62.5) 40 (36.7) 69 (19.8) 3 (15.0)	9 (37.5) 69 (63.3) 280 (80.2) 20 (85.0)	5.02 [1.40–17.40] 2.19 [0.68–7.09] 1.44 [0.45–4.57] 1	0.011 0.19 0.54	1.46 [0.31–6.88] 1.66 [0.46–5.98] 1.29 [0.39–4.24] 1	0.63 0.44 0.68
Fifth-minute APGAR score						
<7 ≥7	41 (38.7) 86 (21.6)	65 (61.3) 313 (78.4)	1.86 [1.28–2.70] 1	0.001	1.44 [0.95–2.18] 1	0.085
Breastfeeding initiated within 1 hour of birth						
Yes No	5 (9.8) 122 (26.9)	46 (90.2) 332 (73.1)	1 2.50 [1.02–6.09]	0.046	1 1.63 [0.64–4.18]	0.31
Neonates who had respiratory distress syndrome						
Yes No	93 (31.5) 34 (16.2)	202 (68.5) 176 (83.8)	1.90 [1.29–2.82] 1	0.001	1.52 [1.01–2.29] 1	0.045

Neonates who had neonatal sepsis						
Yes No	51 (33.8) 76 (21.5)	100 (66.2) 278 (78.5)	1.62 [1.14–2.31] 1	0.008	1.71 [1.18–2.49] 1	0.005
Neonates who had perinatal asphyxia						
Yes No	17 (42.5) 110 (23.7)	23 (57.5) 355 (76.3)	1.99 [1.19–3.32] 1	0.009	2.44 [1.33–4.49] 1	0.004
Neonates who had jaundice						
Yes No	45 (36.9) 82 (21.4)	77 (63.1) 301 (78.6)	1.63 [1.13–2.34] 1	0.009	1.44 [0.99–2.10]	0.057
Neonates who received kangaroo mother care						
Yes No	22 (14.2) 105 (30.0)	133 (85.8) 245 (70.0)	0.43 [0.27–0.68] 1	<0.001	0.48 [0.30–0.77] 1	0.002

Abbreviations: AHR, adjusted hazard ratio; APGAR, appearance, Pulse, Grimace, Activity, and Respiration; CHR, crude hazard ratio; CI, confidence interval; CPAP, continuous positive airway pressure; C/S, caesarean section; GA, gestational age; HIV/AIDS, Human Immunodeficiency Virus/Acquired Immunodeficiency Syndrome; IQR, interquartile range; JUMC, jimma university medical center; NGT, nasogastric tube; NICU, neonatal intensive care unit; PPROM, preterm premature rupture of membrane; SDGs, Sustainable Development Goals; SSA, Sub-Saharan Africa; SVD, spontaneous vaginal delivery.

## Discussion

This study showed that 25.1% of preterm neonates died during the follow-up with an overall neonatal mortality rate of 28.9 deaths per 1,000 neonate days. This finding is consistent with studies reported from Nigeria 27.7% [29], Tigray Region 32.1% with an incidence of 36.6 per 1,000 person days [14], Gondar 32.9 deaths per 1,000 neonate-days [15], and Addis Ababa 25.3% and 36.4 deaths 1,000 neonates-day [12, 30]. However, this finding is higher than studies reported from Australia 7.7% [31], China 1.9% [32] and Uganda 8% [11]. This discrepancy between studies might be explained by variation in a study setting as there might be a high quality of neonatal care in Australia and China. A study from Australia was conducted in a hospital with a level four NICU while this study was conducted in a hospital with a level three NICU. Preterm neonates born in developed countries like Australia and China might receive improved care during pre-pregnancy, pregnancy, antepartum, and postnatal periods. Partly, this disparity might result from a difference in sample size, study design, and those reported studies were multicenter studies. Conversely, this study finding is lower than studies reported from India 33.5% [33], Southern Ethiopia 47.7 deaths per 1,000 neonatal days [17], Mizan Tepi 62.15 deaths per 1,000 neonate-days [13] and Jimma, Ethiopia 34.9% [28]. This discrepancy might result from variation in study design as a study reported from India was a multicenter prospective study conducted on a large sample size. The difference could result from variations in the study settings. The inconsistency with the finding from Jimma might be due to variation in the timing of the study as there was some improvement in antenatal and delivery care from a skilled provider and institutional delivery [22]. Partly, this might result from the fact that the NICU is organized in a good manner, and access to trained healthcare providers increased comparatively since special attention was given to preterm neonates by national neonatal and child survival strategy [21]. This finding implies that ongoing commitment and interventions need to be considered by focusing preterm neonatal survival intervention/management more on the intrapartum, immediate postpartum as well as early neonatal periods.

In this study, early NMR (40 per 1,000 neonate-days) was higher as compared to late NMR (11.7 per 1,000 neonate-days). This finding is consistent with a study reported from Gondar, Ethiopia [15]. This might be attributed to the reason that most of the preterm neonatal mortality in the resource-limited setting is related to practice during the intrapartum and immediate postpartum period, the need for intensive medical care, and timely referral of high-risk neonates. But, this finding is lower than the study conducted in Addis Ababa [30]. This inconsistency could be due to a study from Addis Ababa was a multicenter prospective study conducted on a small sample and it is a setting that receives high-risk neonates referred from different regions of the country. The finding of this study shows the need to focus preterm neonatal survival interventions more on the intrapartum as well as the immediate postpartum period, and early neonatal periods.

In the current study, preterm neonates born from mothers who used antenatal steroids had a lesser hazard of mortality than those neonates born from mothers who did not use antenatal steroids. This finding is in line with studies reported in the United States [34] and China [32]. This could be explained by the fact that the administration of steroids for mothers who had imminent preterm delivery enhances fetal lung maturity and decreases the risk of developing respiratory distress syndrome and intraventricular hemorrhage, and consequently might reduce the risk of neonatal death [35]. In this study, preterm neonates born from mothers who had an obstetric complication during their current pregnancy had a higher hazard of neonatal death compared to their counterparts. This finding is comparable with studies reported from Addis Ababa [12] and Southern Ethiopia [17]. This might be explained by the fact that obstetric complications affect the pregnancy status and placental blood transfusion, and can result in preterm delivery with subsequent preterm-related life-threatening complications which might increase the hazard of neonatal death.

In this study, an increment in gestational age at birth by 1 week decreases the hazard of preterm neonatal deaths by 19%. This finding is in line with studies reported from Gondar [15], and Addis Ababa, Ethiopia [30]. A possible reason for this might be as the gestational age of the neonates at birth increases, the maturity of the fetus will be enhanced, and the risk of developing life-threatening complications related to prematurity decreases which might contribute to a reduced risk of preterm neonatal death.

In the current study, preterm neonates who had RDS had a greater hazard of neonatal mortality compared to their counterparts. This finding is consistent with studies reported from different parts of Ethiopia: Bahir Dar [18] and Jimma [28]. This might be because of similarities in settings that lack postnatal surfactant administration. Partly, it could be explained by the fact that preterm neonates had immature lungs, and might consequently develop life-threatening complications like respiratory failure. Different literatures reported that RDS was the primary cause of preterm neonatal death [36].

In this study, preterm neonates who had neonatal sepsis had a higher hazard of neonatal mortality than preterm neonates without neonatal sepsis. This finding is in line with a study reported from Jimma [28]. This might result from the fact that preterm neonates were more likely to be born with or acquire an infection because they had immature immune defences supplemented with poor calorie intake, which might increase the risk of death [37].

In the current study, preterm neonates who had PNA had a greater hazard of neonatal mortality than those preterm neonates without PNA. This finding is consistent with studies reported from China [32], Gondar [15], Bahir Dar [18], and Jimma, Ethiopia [28]. This consistency might be elucidated by similarity in study design and follow-up period. This finding might be supported by the fact that PNA can lead to hypoxia with subsequent development of acidosis, leading to hypotension and hypoxic-ischemic encephalopathy, which further compromise oxygen delivery to the brain and might increase the risk of death [38].

In the present study, a preterm neonate who received KMC had a 52% lesser hazard of neonatal mortality compared to preterm neonates who did not receive KMC. This finding was in line with studies reported from Uganda [11], Gondar [15], and Bahir Dar, Ethiopia [18]. This consistency might be due to the similarity of the study setting, study design, and sample size. The finding was reaffirmed by the fact that receiving KMC protects neonates from the risk of hypothermia by decreasing body surface area to the external environment. Partly, it might also be explained by the fact that KMC promotes early initiation of breastfeeding, and may be used even when babies on formula-fed, which helps to prevent hypoglycemia. Moreover, KMC helps to reduce neonatal mortality by protecting them from sepsis [39, 40].

This study has some limitations. Since the data were accessed from a secondary source, some important predictors such as maternal educational status, maternal nutritional status, birth interval, birth order, duration of rupture of membrane, and first-minute APGAR score were not available in the medical records and their effect on preterm neonatal mortality was not investigated. The study did not address the probable care and service-related predictors of mortality among preterm neonates due to the nature of the study design. Additionally, the study covers only JUMC which limits generalizability to other settings found in the Oromia region and Ethiopia.

### Conclusion

The incidence rate of preterm neonatal mortality was found high. Most preterm neonatal mortality occurs in the early phase of the neonatal period, which requires due attention to meet the national newborn and child survival and SDG-3 in Ethiopia. Obstetric complications, respiratory distress syndrome, neonatal sepsis, and perinatal asphyxia were found to be predictors of preterm neonatal mortality. Whereas, antenatal steroid exposure, an increment in gestational age at birth, and receiving kangaroo-mother care were preventive predictors for preterm neonatal mortality. Hence, special emphasis and close follow-up are highly warranted, especially during the early neonatal period. It is better to strengthen obstetrics and use of antenatal steroids for women having an imminent preterm delivery. Early detection and management of obstetric as well as preterm neonatal complications is highly demanding. Encouraging and supporting mothers to practice kangaroo-mother care, and ensuring a continuum of care are also crucial to enhance preterm neonatal survival.

## Supplementary Material

SUPPLEMENTARY MATERIAL S1
A flow chart of selecting preterm neonates admitted to the NICU of JUMC, Southwest Ethiopia, 2020.
