# Supplementary figures and images for: Incidence and Predictors of Mortality Among Preterm Neonates Admitted to Jimma University Medical Center, Southwest Ethiopia: a Retrospective Follow-Up Study
Source: Int J Public Health. 2024 Jul 4;69:1606897. doi: 10.3389/ijph.2024.1606897 (PMC11255349; doi:10.3389/ijph.2024.1606897)

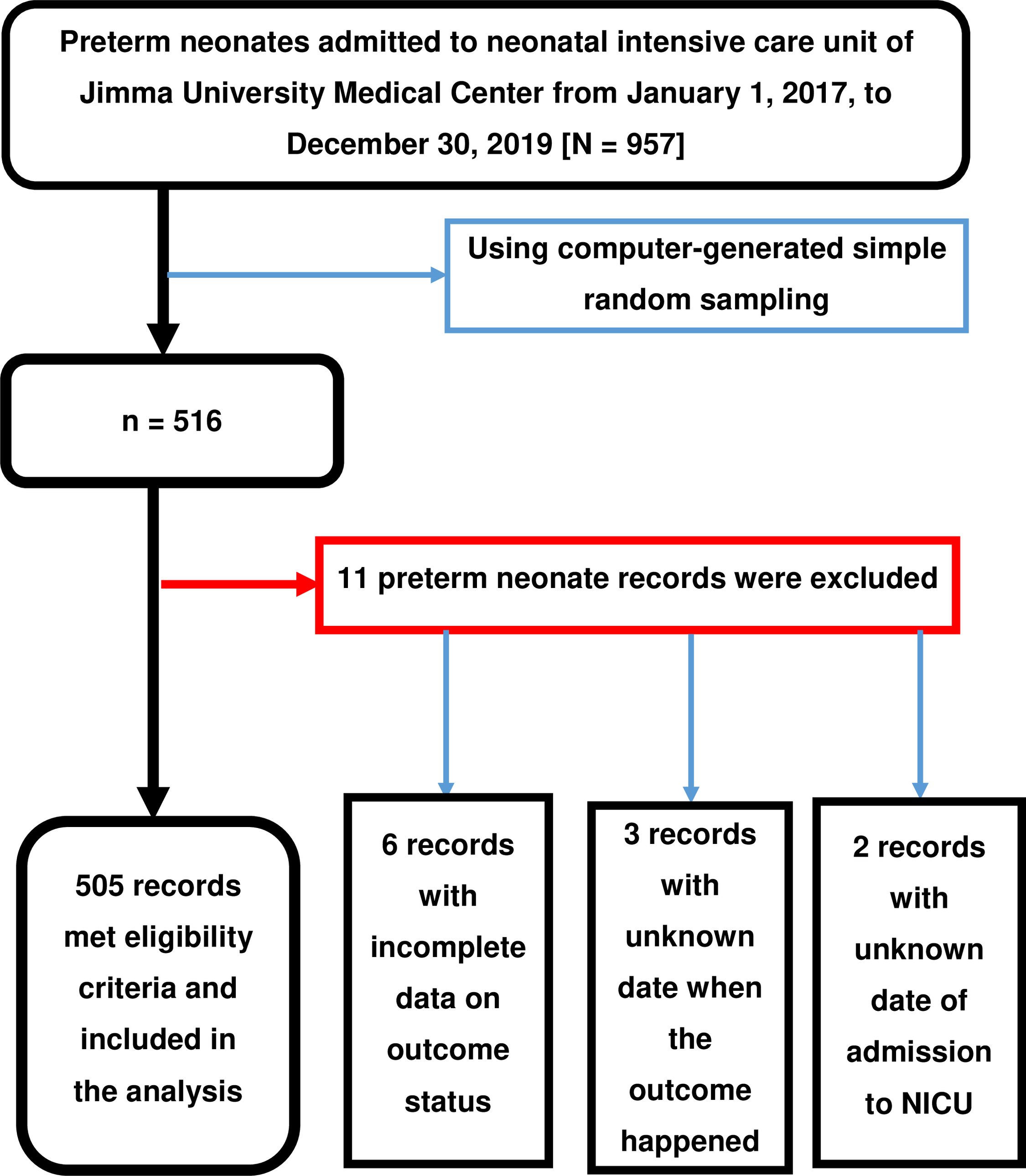

Supplement: Supplementary file 1 [file Image1.TIF]
